# Clinical Impact of Colistin Banning in Food Animal on *mcr-1*-Positive Enterobacteriaceae in Patients From Beijing, China, 2009–2019: A Long-Term Longitudinal Observational Study

**DOI:** 10.3389/fmicb.2022.826624

**Published:** 2022-02-10

**Authors:** Qian Zhao, Yiming Li, Yingxin Tian, Yueyun Shen, Shaolin Wang, Ying Zhang

**Affiliations:** ^1^Department of Laboratory Medicine, The First Medical Center, Chinese PLA General Hospital, Beijing, China; ^2^College of Veterinary Medicine, China Agricultural University, Beijing, China

**Keywords:** colistin, *mcr-1*, Enterobacteriaceae, longitude study, China

## Abstract

The colistin resistance gene *mcr-1* is emerging as a global public health concern, altering the regulation of colistin usage globally since 2017, especially in China. However, few studies have revealed the impact of policy change on the epidemiology of *mcr*-positive Enterobacteriaceae (MCRPE) in patients. Here, we describe a molecular epidemiological study to investigate the MCRPE in patients in China from 2009–2019. During the surveillance period, 26,080 non-duplicated Enterobacteriaceae isolates were collected in Beijing. Colistin-resistant isolates were screened by enrichment culture supplemented with colistin, and the presence of the *mcr* gene was determined by PCR amplification. MCRPE isolates were then analyzed by susceptibility testing, genotyping, and risk factor analysis. Of the 26,080 isolates, *mcr-1* was detected in 171 (1.1%) of 15,742 *Escherichia coli* isolates and 7 (0.1%) of 10,338 *Klebsiella pneumoniae* isolates. The prevalence of *mcr-1*-positive *E. coli* (MCRPEC) showed an increasing trend from 2009 to 2016, while a decreasing trend was observed since 2017. Multi-locus sequence typing analysis showed that MCRPEC isolates had extremely diverse genetic backgrounds, and most of these isolates were non-clonal. The prevalence of MCRPE in China remained at a low level, and even showed a declining trend over the last 3 years after the banning of colistin usage as feed additive in food animal in 2017. However, colistin permission in clinical therapy could still increase the risk of MCRPE transmission and intractable infections, active surveillance and monitoring strategies of MCRPE are recommended to prolong the clinical longevity of colistin.

## Introduction

Colistin is regarded as one of the last therapeutic options available to treat infections caused by carbapenem-resistant Enterobacteriaceae (CRE), which pose an increasing risk to public health. Until 2015, resistance to colistin was only associated with mutations and regular changes in chromosomal genes (Wang et al., 2018). Liu et al. (2016) described a plasmid-mediated colistin resistance gene, *mcr-1*, which encodes a phosphoethanolamine transferase enzyme (MCR-1) and results in the addition of phosphoethanolamine to lipid A in Enterobacteriaceae. The emergence of the mobile colistin resistance gene *mcr-1* could result in bacterial isolates being resistant to all classes of antibiotics, which will compromise the available treatment options for severe infections (Zhong et al., 2018).

Enterobacteriaceae, especially *Escherichia coli* and *Klebsiella pneumoniae*, typically form part of the normal flora in healthy people, yet can induce various infections under certain conditions, such as respiratory, urinary, and bloodstream infections (Paterson, 2006; Seiffert et al., 2013). Although the use of colistin in humans has been very limited in the past, it has been implemented in veterinary medicine since the early 1980s, mainly for the prevention and treatment of Enterobacteriaceae infections (Kempf et al., 2016). In China, colistin sulfate premix has been widely used as animal feed additive since 2009, and the production has reached 30,000 tons in 2015 (Wang et al., 2020). Wang et al. (2017) speculated that the emergence of *mcr-1* probably occurred first in animals, before extending to humans. Liu et al. (2016) identified the plasmid-borne colistin resistance gene, *mcr-1*, in Enterobacteriaceae from hospitalized humans, animals, and raw meat from China in 2015 (Wang et al., 2017). Subsequently, *mcr-1*-positive Enterobacteriaceae (MCRPE) have been found in inpatients, healthy humans, animals, raw meat, vegetables, and a number of environmental settings globally (Quesada et al., 2016). *E. coli* and *K. pneumoniae* are considered key reservoirs and disseminators of *mcr-1*. On April 30, 2017, the Chinese Ministry of Agriculture formally issued the banning of colistin as feed additives for food animals, aiming to reduce the wide spread of colistin resistance and protect the effectiveness of colistin in the clinical setting.

To date, MCRPE have been reported in numerous countries across Asia, Africa, Europe, North America, and South America (Quan et al., 2017; Saavedra et al., 2017; Wang et al., 2018; Clemente et al., 2019), indicating the rapid transfer of *mcr-1* among Enterobacteriaceae (Carattoli, 2013). Colistin has been approved for human medicine by the China Food and Drug Administration since January 2017 (Shen Y. et al., 2018), there is an urgent need to assess the role of MCRPE in the treatment of infections. Herein, we investigated the prevalence, risk factors, and molecular epidemiology of MCRPE carriage among inpatients and outpatients in Beijing, the capital of China, from 2009 to 2019. The aim of our study was to better understand the clinical impact of policy change on the current epidemiological trends and characteristics of MCRPE colonization in patients over this time frame.

## Materials and Methods

### Study Design

The aim of this epidemiological and clinical study was to investigate the prevalence of MCRPE in patients. We conducted a long-term molecular epidemiological surveillance in Beijing from 2009–2019. Beijing, the capital city of China, has a population of 21 million and widely accepts patients from throughout the country. MCRPE strains were isolated from different specimen types in patients, including blood, urine, sputum, fecal, and tissue. At least 1,000 samples were collected annually.

All samples were taken from non-duplicate patients and we screen MCRPE isolates from samples by culturing in enrichment media supplemented with 2 mg/L colistin. The isolates before 2017 were identified from the retrospective collection. Pure colonies of *E. coli* and *K. pneumoniae* were selected according to their morphology and color on an SS agar plate supplemented with 2 mg/L colistin. The surviving bacteria were identified by matrix-assisted laser desorption/ionization time of flight mass spectrometry (MALDI-TOF MS) (Bruker Daltonik GmbH, Bremen, Germany), and further confirmed by 16S rDNA sequence analysis. Ethics approval was granted by the Chinese PLA General Hospital. Individual consent forms obtained from all patients before sampling. All participants held the right to quit the study at any stage.

### Genetic Screening and Sequence Type Analysis

The presence of the colistin-resistance genes (*mcr*) was confirmed by Sanger sequencing (Seiffert et al., 2013). All *mcr-*positive strains were subjected to whole-genome sequencing (WGS). Genomic DNA extraction of isolates was performed using the Wizard Genomic DNA Purification Kit (Promega, Beijing, China) following a standard protocol, and then sequenced on the Illumina HiSeq 2500 platform (Annoroad Biotec Co.) with a 150-bp paired-end strategy. All procedures were carried out according to the manufacturer’s instructions. The sequencing data were analyzed using multiple programs. The draft assembly of the sequences was generated using SPAdes version 3.11.1. Colistin resistance gene *mcr* and multi-locus sequence typing (MLST) were confirmed using standalone BLAST analysis SRST2 (Shen Z. et al., 2018).

### Antimicrobial Susceptibility Testing

We calculated minimum inhibitory concentrations (MICs) for all MCRPE isolates against commonly used antibiotics *via* the broth microdilution method in accordance with the Clinical and Laboratory Standards Institute (CLSI) guidelines. Breakpoints for aminoglycosides (amikacin, gentamicin, and tobramycin), β-lactams (ampicillin and piperacillin), β-lactam/β-lactamase inhibitor combination (amoxicillin-clavulanate, ampicillin-sulbactam, and piperacillin-tazobactam), carbapenems (ertapenem, imipenem, and meropenem), cephems (cefazolin, cefepime, ceftazidime, cefotetan, and ceftriaxone), fluoroquinolones (ciprofloxacin and levofloxacin), trimethoprim-sulfamethoxazole, and nitrofurantoin were interpreted according to the annual CLSI-M100-S28^1^ [Clinical and Laboratory Standards Institute (CLSI), 2018], while the results of antimicrobial susceptibility for colistin was interpreted in accordance with the European Committee on Antimicrobial Susceptibility Testing (EUCAST) criteria^2^ [European Committee on Antimicrobial Susceptibility Testing [ECAST], 2020]. The reference strains *E. coli* ATCC 25922 and *K. pneumoniae* ATCC 13883 (carbapenem-susceptible) were used as controls.

### Classification of Variants and Statistical Analysis

We collected clinical data from patients with or without *mcr*-positive isolates between 2009 and 2019. Multiple risk factors were assessed, including sex (male and female), age (<15, 15–24, 25–34, 35–44, 45–54, 55–64, and ≥ 65 years), living conditions (city or village), specimen types (blood, sputum, and urine), patient types (inpatient or outpatient), comorbidities (diabetes, hypertension, and malignant tumor), and other risk factors (operation history, mechanical ventilation, urinary catheter, drainage tube, venous catheterization, and antibiotic use in the past 3 months). Variants were collated into specifically designed databases using Microsoft Excel 2016 (Microsoft, Redmond, WA, United States). Univariate analysis was performed using the Statistical Package for the Social Sciences version 23.0 (SPSS, Chicago, IL, United States). A chi-square test was used to examine the difference in the resistance levels of the different groups, and variables with a *p*-value < 0.2 were considered statistically significant. Significant variables with *P* < 0.2 were taken into multivariable analysis. A multivariable logistic regression model using a backward stepwise process was adopted to estimate the odds ratios (ORs) and 95% confidence intervals (CIs) of the risk factors related to *mcr*-positive *E. coli.* Variables with a *p*-value < 0.05 were considered significant risk factors. Pooled prevalence was estimated using a metaphor package with 95% CIs to represent the resistance rates of bacteria from 2010 to 2019 (Zou et al., 2019).

## Results

### Overview of MCRPE Strains

**FIGURE 1:**
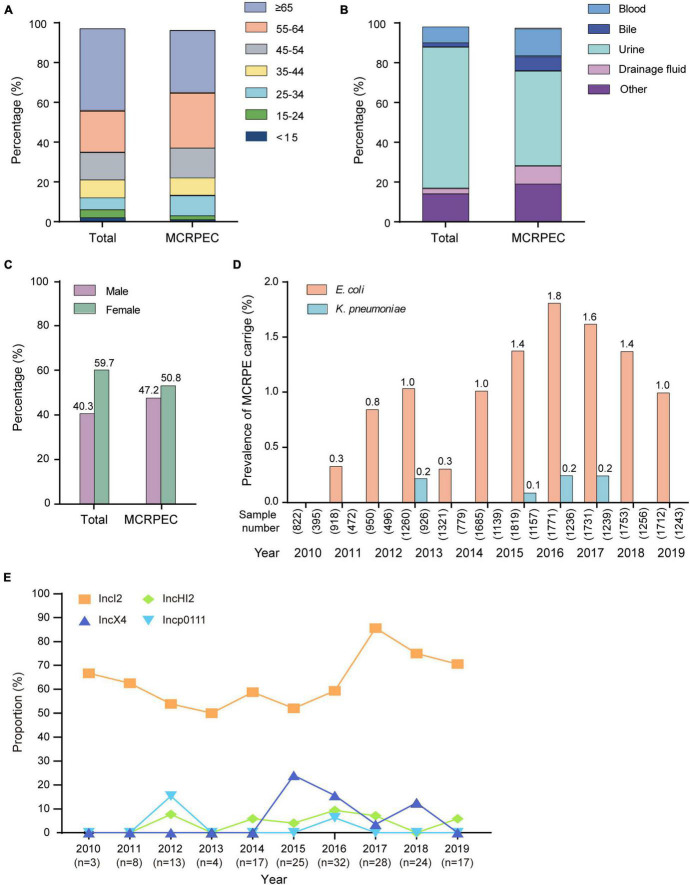
Summary of information. **(A)** The age distribution of the total sample population and *mcr-1*-positive *E. coli* (MCRPEC). **(B)** The specimen type distribution of the total sample population and MCRPEC. **(C)** The sex distribution of the total sample population and MCRPEC. **(D)** The prevalence of *mcr-1*-positive Enterobacteriaceae (MCRPE) during 2009–2019. **(E)** The prevalence of main plasmids types in all MCRPEC during 2010–2019.

For the prevalence study, a total of 26,080 non-duplicated isolates from inpatients and outpatients were collected in Beijing from 2009 to 2019, which included 15,742 *E. coli* and 10,338 *K. pneumoniae* isolates. We detected the *mcr-1* in 171 (1.09, 95 CI, 0.93–1.26) *E. coli* (MCRPEC) and 7 (0.07, 95 CI, 0.03–0.14) *K. pneumoniae* (MCRPKP) isolates (Supplementary Table 1). The age, specimen type, and sex information about the MCRPEC cases and the study population are shown in Figures 1A–C, and the information of the 7 MCRPKP cases is shown in Supplementary Table 2. The majority of the patients were over 65 (41.4%), and most isolates were obtained from urine (71.1%). The distribution of age, sex, and specimen information was similar among the MCRPEC cases and total cases. During the study period, the overall percentage of *mcr-1*-positive isolates remained low, especially for *K. pneumoniae*, and we found diverse trends in the prevalence of MCRPEC (Figure 1A and Supplementary Table 1). The proportion of MCRPEC increased from 2009 (0.0%) to 2016 (1.8%) (*p* < 0.001), except in 2013 (0.3%), while a decreasing trend was observed since 2017 (1.6%) (Figure 1A).

### Antimicrobial Resistance Patterns of MCRPE

Overall, 150/171 (87.72%) MCRPEC and all 7 MCRPKP strains exhibited resistance to colistin (Supplementary Tables 3, 4). Antimicrobial susceptibility profiles of the 171 MCRPEC strains are shown in Supplementary Table 3. Among the 171 MCRPEC isolates, the MICs for colistin ranged from 0.25 to 16 μg/mL. Most MCRPEC remained susceptible to amikacin (85.4%), ertapenem (95.9%), imipenem (98.2%), piperacillin-tazobactam (91.0%), and cefotetan (96.2%). Among the 171 MCRPEC isolates, 3 (1.8%) were carbapenem-resistant, 132 (77.2%) were extended-spectrum β-lactamases (ESBL), and the proportion of ESBL strains increased from 50.0% in 2010 to 82.4% in 2019 (data not shown). We classified the MCRPEC isolates from 2010 to 2019 into inpatient and outpatient groups. The MIC_50_ values were similar between clinical *E. coli* isolates from inpatients (*n* = 127) and outpatients (*n* = 44), except for gentamicin and amikacin, which were both more than 16-fold higher in the inpatient group (Supplementary Table 5).

### Risk Factors and Associated Outcomes of MCRPEC Infection

We collected clinical data from 171 *mcr-1*-positive clinical *E. coli* isolates and randomly selected 734 *mcr-1*-negative clinical *E. coli* isolates (62 isolates were excluded because of incomplete data, leaving 672) from 15,571 *mcr-1*-negative *E. coli* infection cases. Multiple variables were assessed to determine the risk factors associated with MCRPEC by OR analysis (Tables 1, 2). Age and living conditions were not associated with MRCPEC infection. We determined that *E. coli* isolated from male were more likely to be *mcr-1* positive compared to those from outpatients (OR = 1.6, *p* < 0.02). Specimen types of *E. coli* were significantly associated with *mcr-1* positivity; *E. coli* isolates from bile and drainage fluid samples had a higher proportion of MCRPEC than those from other samples. Furthermore, MCRPEC was far more prevalent among *E. coli* isolated from patients with malignant tumors (*R* = 2.3, *p* < 0.001).

**TABLE 1:** Analysis of risk factors associated with *mcr-1*-positive *E. coli* (*n* = 843).

Variables	*mcr-1*-positive *E. coli* (*n* = 171)		*mcr-1*-negative *E. coli* (*n* = 672)		OR (95% CI) ^a^	*P* ^b^
Sex						0.051
Female	89	52.0%	405	60.3%	1.0	
Male	82	48.0%	266	39.6%	1.4 (1.0–2.0)	
Age						
<15	1	0.6%	19	2.8%	0.2 (0.0–1.5)	**0.150**
15–24	4	2.3%	27	4.0%	0.6 (0.2–1.7)	0.298
25–34	18	10.5%	42	6.3%	1.8 (1.0–3.2)	0.052
35–44	17	9.9%	65	9.7%	1.0 (0.6–1.8)	0.916
45–54	27	15.8%	95	14.1%	1.1 (0.7–1.8)	0.583
55–64	49	28.7%	145	21.6%	1.5 (1.0–2.1)	0.05
≥65	55	32.1%	279	41.5%	0.7 (0.5–1.0)	**0.026**
Living condition						0.683
City	141	82.5%	566	84.2%	1.0	
Village	29	17.0%	106	15.7%	1.7 (0.7–1.7)	
Treatment						**0.029**
Outpatient	41	24.0%	219	32.6%	1.0	
Inpatient	130	76.0%	453	67.4%	1.5 (1.0–2.3)	
Specimen type						
Blood	23	13.3%	105	15.6%	1.0	0.429
Urine	79	46.4%	373	55.5%	1.0 (0.6–1.6)	**0.003**
Bile	8	4.4%	12	1.8%	5.0 (2.2–11.1)	**<0.001**
Drainage fluid	11	6.6%	11	1.7%	6.7 (3.0–14.6)	**<0.001**
Comorbidities and risk factors						
Diabetes	17	9.9%	123	18.3%	1.0	**0.009**
Hypertension	37	21.6%	193	28.7%	1.4 (0.8–2.6)	0.063
Malignant tumor	46	26.9%	92	13.7%	3.6 (2.0–6.7)	**<0.001**
Operation history	70	40.9%	216	32.1%	2.3 (1.3–4.2)	**0.030**
Mechanical ventilation	9	5.3%	52	7.7%	1.3 (0.5–3.0)	0.265
Urinary catheter	35	20.5%	179	26.6%	1.4 (0.8–2.6)	0.098
Drainage tube	36	121.1%	160	23.8%	1.6 (0.9–3.0)	0.446
Venous catheterization	25	14.6%	104	15.5%	1.7 (0.9–3.4)	0.781
Antibiotic use in the past 3 months	65	38.0%	158	23.5%	3.0 (1.7–5.3)	**<0.001**

*^a^CI, confidence interval; OR, odds ratio. ^b^P < 0.05 are shown in boldface.*

**TABLE 2:** Multivariable logistic regression analysis of factors associated with *mcr-1*-positive *E. coli*.

Variables	OR (95% CI) ^a^	*P* ^b^
Sex		**0.023**
Female	1	
Male	1.6 (1.1–2.4)	
Specimen type		
Blood	1.0	NA
Urine	2.0 (1.1–3.4)	**0.020**
Bile	4.2 (1.5–12.0)	0.007
Drainage fluid	6.0 (2.2–16.0)	**<0.001**
Comorbidities and risk factors		
Diabetes	0.5 (0.3–0.8)	**0.007**
Malignant tumor	2.3 (1.5–3.6)	**<0.001**
Urinary catheter	0.5 (0.3–0.7)	**0.001**

*^a^CI, confidence interval; OR, odds ratio. ^b^P < 0.05 are shown in boldface.*

In addition, we analyzed the outcome of patients in 28 days with MRCPEC or *mcr-1*-negative *E. coli* (Table 3). Significant differences were observed between the two groups; in particular, patients with MRCPEC infections were more likely to result in treatment failure.

**TABLE 3:** Patient outcomes with *mcr-1*-positive *E. coli* and *mcr-1*-negative *E. coli* infections.

Variables	*mcr-1*-positive *E. coli* (*n* = 120)	*mcr-1*-negative *E. coli* (*n* = 504)	OR (95% CI) ^a^	*p* ^b^
Cure	5 (4.2%)	76 (15.1%)	0.2 (0.1–0.6)	**0.001**
Improve	103 (85.8%)	405 (80.4%)	1.5 (0.8–2.6)	0.166
Treatment failure	12 (10.0%)	23 (4.6%)	2.3 (1.1–4.8)	**0.020**

*^a^CI, confidence interval; OR, odds ratio. ^b^P < 0.05 are shown in boldface.*

### Molecular Epidemiology of MCRPEC

**FIGURE 2:**
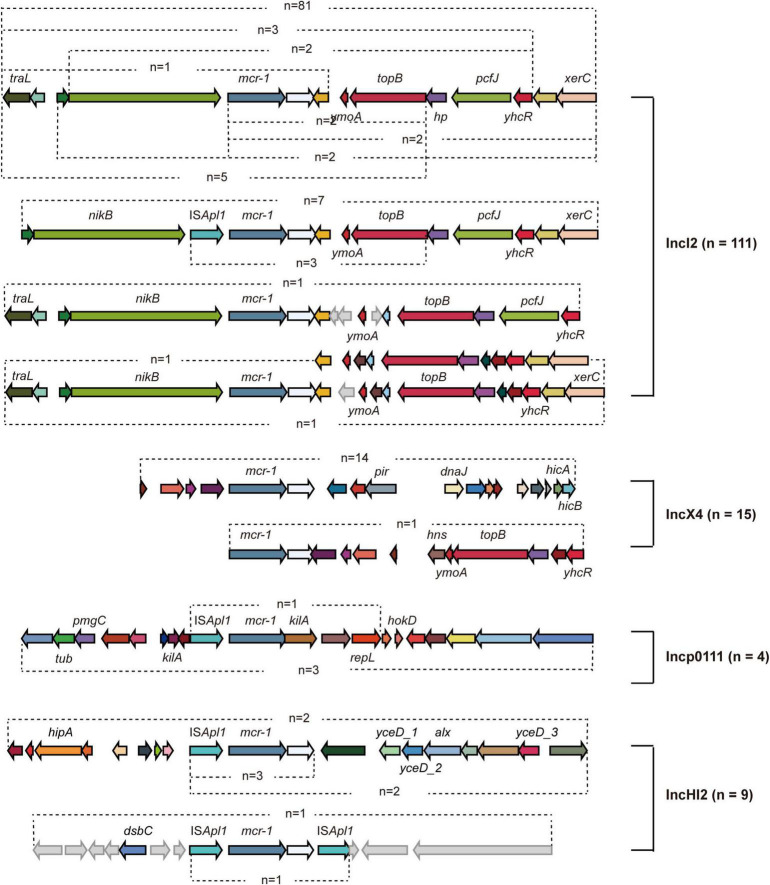
Genetic analysis *of mcr-1*- carrying plasmids. Different arrows represent particular genes having a name written underneath of each arrow. The arrows without names represent hypothetical proteins.

**FIGURE 3:**
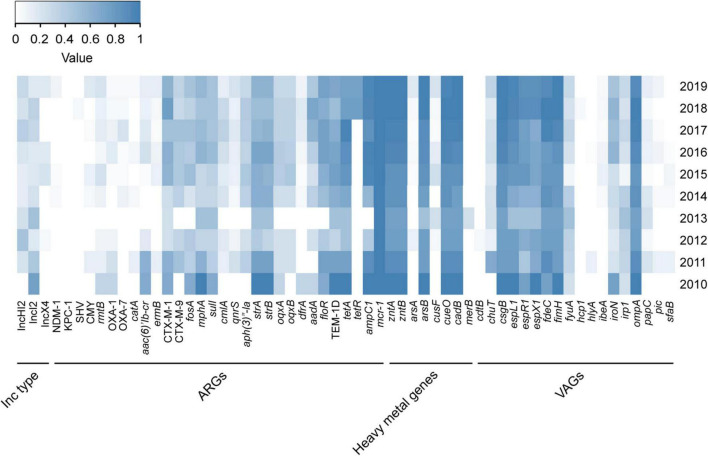
Distribution of Inc type, ARGs, heavy metal genes, and VAGs among MCRPEC isolates. The color of each box represents the percentage of the corresponding item among sequenced isolates in the corresponding year. ARGs, antibiotic resistance genes; HMGs, heavy metal genes; VAGs, virulence-associated genes.

We further evaluated the molecular characteristics of MRCPEC strains as minimum spanning trees, phylogenetic tree and heatmaps (Figures 2, 3 and Supplementary Figures 1, 2). There were 66 distinct sequence types (STs) in the 171 isolates, suggesting extreme divergence of MRCPEC strains. Molecular epidemiological analysis revealed that none of these STs were predominant, but ST410 (*n* = 10), ST648 (*n* = 9), ST156 (*n* = 7), and ST224 (*n* = 7) were more prevalent than the other STs. These four STs have been recognized as common clades of *E. coli* carrying *mcr-1* in a previous study (Wang et al., 2017). Our results suggest that horizontal dissemination of *mcr-1* in *E. coli* was discovered between 2014 and 2019. In addition, an ST617 *E. coli* isolate in 2014 and an ST3224 *E. coli* isolate in 2015 carried both *mcr-1* and the carbapenem resistance gene *bla*_NDM–1_.

**FIGURE 4:**
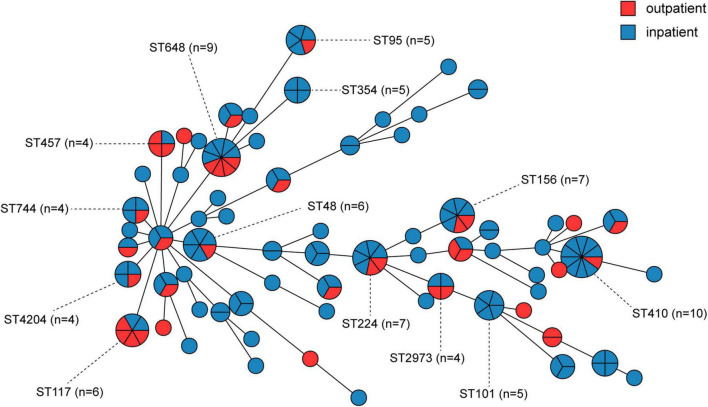
Minimum spanning tree of *mcr-1-*positive *E. coli* by MLST type and gene allele from inpatients and outpatients. Each node in the tree represents an ST, and the size of a node is proportional to the number of isolates it represents. The length of the branch is equal to the number of different alleles (calculated using seven MLST genes) between two linked nodes.

In order to investigate the correlation between human MCRPEC and animal MCRPEC, the whole genomes of 150 animal MCRPEC, 122 animal *mcr-1* negative *E. coli* (MCRNEC), and 137 human MCRNEC were downloaded from the published data in the National Center for Biotechnology Information (NCBI) database. All the MCRPEC and MCRNEC isolates were allocated to 142 STs, and the diversity of clinical origin was greater than isolates of animal origin (Supplementary Figure 1). Of these *mcr-1*-positive clades, most sequence-types were common to both animal and human origin, such as ST156, ST101, ST354, and ST48 (Figure 4 and Supplementary Figure 1). Interestingly, several ST branches were discovery only in human carriage, such as ST131 and ST95 (Supplementary Figure 1).

A total of 139 *mcr-1* positive plasmids were identified and assigned to known Inc types, among which the most prevalence was IncI2 (*n* = 111, 64.9%), followed by IncX4 (*n* = 15, 8.8%), IncHI2 (*n* = 9, 5.3%), and Incp0111 (*n* = 4, 2.3%). IncI2 remains the dominant Inc type in MCRPEC prevalence during 2010 to 2019, while a decreasing trend was observed in the proportion of IncI2 plasmids from 2017 to 2019 (Figure 1E). The genetic environment of *mcr-1* depends on its backbone structure plasmids, which vary greatly between different Inc types (Figure 2). The genetic context of *mcr-1* within each plasmid type (IncI2, IncX4, IncHI2, and Incp0111) was similar to the four reported *mcr-1*-carrying plasmid (pHNSHP45, KX254343, MF774186, and MF455226, respectively) from four pig-derived *E. coli* isolates (Supplementary Figures 3–6). Notably, the ancestral mobile element, ISApl1, which previously thought to be responsible for *mcr-1* transmission, was only detected in 42 (24.6%) isolates.

Antibiotic resistance genes (ARGs), heavy metal genes, and virulence-associated genes (VAGs) in the 171 MCRPEC isolates were determined by WGS. The percentages of ARGs, heavy metal genes, and VAGs for each year are shown in Figure 3. The *mcr-1* gene co-existed with *strA/B* (aminoglycoside resistance), *ampC1* (β-lactam ARG), *bla*_CTX–M–1_ and *bla*_TEM–1D_ (ESBL-encoding genes), *floR* (florfenicol resistance), and *tetA* (tetracycline resistance gene) (Figure 3). *espL/R/X* (type III secretion system), *csgB* (which encodes the curli nucleator protein of *E. coli*), *ompA* (outer membrane protein A), *fdec* (intimin-like protein), and *fimH* (type I fimbriae) were more strongly associated with *mcr-1* carriage than other VAGs (Figure 3). Furthermore, WGS showed that *mcr-1* was mostly located on 3 Inc (incompatible) type plasmids, IncX4-type (*n* = 21, 12.3%), IncI2-type (*n* = 115, 67.3%), and IncHI2-type (*n* = 28, 16.4%), which is similar to a previous report (Jiang et al., 2020).

## Discussion

In 2009–2016, we noted a significant increase in the prevalence of MCRPEC from 2009 to 2016 (0.0% ∼ 1.8%), except in 2013, while a decreasing trend was noted after 2016 (Figure 1D). Notably, the Ministry of Agriculture of China (Article number 2428) withdrew colistin as a feed additive and growth promoter in November 2016, and this was officially enforced in April 2017 (Walsh and Wu, 2016). Colistin produced before Apr, 2017 was still allowed to use according to the data from Ministry of Agricultural. The overall production of colistin sulfate reached over 50,000 tons in 2015–2016, we believed the rising of colistin resistance may be later comparing with the sales and usage of colistin, and several studies also indicated colistin resistance reached peak around later 2016-mid 2017 (Shen et al., 2016; Wang et al., 2020). In Tu et al.’s study, the prevalence of *mcr-1* (5.6%) was significantly lower than before the ban (86.4%, *p* < 0.01) in a large scale swine farm (Tu et al., 2021). According to previous study, the banning had a significant effect on reducing colistin resistance in both animal and humans by comparing 2016–2017 and 2018–2019 (Shen et al., 2020; Wang et al., 2020). Furthermore, the human carriage of MCRPEC also decreased from 14.3% in 2016 to 6.3% in 2019 (*p* < 0.0001) in hospital across 24 provincial capital cities and municipalities in China (Wang et al., 2020). In a prevalence dynamics analysis of human *mcr-1* colonization from April 2011 to December 2019, a dramatic decline in human *mcr-1* colonization prevalence was observed, consisting with the complete ban of colistin in animal feed (Shen et al., 2021). Above all, we suggest that the withdrawal of colistin as an animal growth promoter in China had a positive impact on MCRPEC infections in both animals and humans. However, we believed the overall colistin resistance will remain declining as long as the banning in effective. It might take a few extra years for the resistance reduced to the stage before colistin usage. In this study, the prevalence of *mcr-1* decreased from 32 (1.8%) of 1,771 in 2016, to 24 (1.6%) of 1,731 (*p* = 0.3) in 2018 and 17 (1.0%) of 1,712 (*p* < 0.05) in 2019. However, the extended longitude study was necessary to further evaluate the effective of banning.

In the analysis of different factors associated with MCRPEC carriage, we observed that *E. coli* from inpatients were more likely to carry the colistin resistance gene *mcr-1* (OR = 1.5, *p* < 0.05), suggesting that hospitalization may be a risk factor for colonization of *mcr-1*. Among the different specimen types, we observed that *E. coli* isolates from bile and drainage fluid were much more likely to be *mcr-1* positive than from urine and blood. In addition, we observed a significant association between MCRPEC infection and comorbidities, such as diabetes, malignant tumors, and operation history (*p* < 0.05). Unsurprisingly, antibiotic use in the past 3 months was strongly associated with high MCRPEC occurrence.

Our MLST analysis showed that the 171 MRCPEC isolates had extremely diverse genetic backgrounds, and many were non-clonal. In this study, ST410 (*n* = 9) was the most prevalent sequence type among inpatients, while ST648 (*n* = 4) and ST117 (*n* = 4) were more prevalent than other types among outpatients. *E. coli* ST410 has been reported worldwide as an extraintestinal pathogen associated with multidrug resistance and is capable of patient-to-patient transmission, causing hospital outbreaks (Roer et al., 2018). *E. coli* ST648 is a predominant multidrug-resistant clone observed worldwide and is frequently associated with various β-lactamases, including ESBLs, NDM, and KPC (Mushtaq et al., 2011; Kim et al., 2012). *E. coli* ST117 is a highly virulent pathogenic lineage associated with extraintestinal infections in humans and poultry (Cummins et al., 2019). These findings indicate that *mcr-1* has wide host adaptability in *E. coli* and different virulence potentials. Of all *mcr-1*-positive isolates, most sequence-types were common in both human and animal origin, and nucleotide sequences of four main plasmid Inc-types (IncI2, IncX4, IncHI2, and Incp0111) were similar to reported plasmids from animal origins in China, which reminds us the speculation that *mcr-1* probably occurred first in animals and extended to humans (Wang et al., 2017).

The plasmid-mediated gene *mcr-1* has disseminated globally and could pose severe threats to human health when transferred into CRE and extended-spectrum beta-lactamase Enterobacteriaceae (ESBL-E), leaving fewer treatment options for infections caused by multi-drug resistant strains. Among 178 *mcr-1* positive Enterobacteriaceae, 161 (90.5%) isolates harbored ESBL-encoding genes (such as *bla*_CTX–M_ and *bla*_TEM_) and 35 (19.7%) isolates were carbapenemases (such as NDM-1, KPC-2, and OXA-48) producer. To date, a few reports showed that 0.1–4.6% CRE strains were *mcr-1* positive in clinical settings (Huang et al., 2018; Chen et al., 2019; Xiaomin et al., 2020), and one study showed that 2.40% ESBL-E strains also harbored *mcr-1* (Jørgensen et al., 2017). The coexistence of transferable colistin and carbapenem resistance has become an alarming concern, calling for global monitoring and surveillance. Although mobile colistin resistance poses great difficulties in the treatment of severe infectious diseases, in this study, most *mcr-1* positive isolates remained susceptible to many other antibiotics, such as carbapenems, piperacillin-tazobactam, amikacin, and cefotetan. Therefore, antimicrobials should be used in combination to defend against colistin-resistant bacteria in clinical therapy.

Our study has several limitations. One limitation was that the colistin resistance gene *mcr-1* was screened in Enterobacteriaceae isolates collected retrospectively. It is possible to lose *mcr-1* carrying plasmids during preservation, which may result in an underestimation of the prevalence of *mcr-1*. Another limitation is the partial collection of patient information. Since the colossal groups surveyed in this study, we only included 672 (4.3%) of 15,571 *mcr-1* negative *E. coli* isolates for multivariable analysis. Even though, our data were large-scale and combined across different periods each year. For further studies, analysis of more isolates should be included to reduce bias in identifying the risk factors related to *mcr-1* in patients.

In conclusion, this survey revealed a decreasing prevalence from 2016 to 2019, which is opposite to the increasing trend observed between 2009 and 2016. MCPREC isolates possessed extremely diverse genetic backgrounds in patients in the clinical setting from Beijing, China, and our findings provide evidence of the transmission of *mcr-1* among patients. Moreover, the coexistence of transferable colistin resistance and carbapenem resistance poses a great threat, and effective monitoring and surveillance are necessary to control and prevent the dissemination of MCRPE.

## Data Availability Statement

Whole-genome sequencing data that support the findings of this study have been deposited in the NCBI database under BioProject accession number PRJNA78361.

## Author Contributions

QZ contributed significantly to perform the experiments. YL contributed significantly to perform the data analyses and wrote the manuscript. YT and YS helped to perform the experiments and manuscript preparation. SW and YZ contributed significantly to the conception of the study. All authors contributed to the article and approved the submitted version.

## Conflict of Interest

The authors declare that the research was conducted in the absence of any commercial or financial relationships that could be construed as a potential conflict of interest.

## Publisher’s Note

All claims expressed in this article are solely those of the authors and do not necessarily represent those of their affiliated organizations, or those of the publisher, the editors and the reviewers. Any product that may be evaluated in this article, or claim that may be made by its manufacturer, is not guaranteed or endorsed by the publisher.

## Footnotes

1
http://www.clsi.org/

2
https://www.eucast.org/
