# Supplementary material for: Clinical Impact of Colistin Banning in Food Animal on mcr-1-Positive Enterobacteriaceae in Patients From Beijing, China, 2009–2019: A Long-Term Longitudinal Observational Study
Source: Front Microbiol. 2022 Feb 10;13:826624. doi: 10.3389/fmicb.2022.826624 (PMC8866948; doi:10.3389/fmicb.2022.826624)
Supplement: Supplementary file 1 [file Data_Sheet_1.PDF]

Supplementary materials

Table S1. Prevalence of MCRPE strains in different years.

Year	*mcr-1*-positive *E. coli*		*mcr-1*-positive *K. pneumoniae*		*P*
	%	95% CI	%	95% CI	
2009	0.0 (0/822)	0.0-0.4	0.0 (0/395)	0.0-0.9	NA[a]
2010	0.3 (3/918)	0.1-0.9	0.0 (0/472)	0.0-0.8	0.555
2011	0.8 (8/950)	0.4-1.6	0.0 (0/496)	0.0-0.8	0.094
2012	1.0 (13/1260)	0.5-1.7	0.2 (2/926)	0.0-0.8	0.032
2013	0.3 (4/1321)	0.1-0.7	0.0 (0/779)	0.0-0.5	0.303
2014	1.0 (17/1685)	0.6-1.6	0.0 (0/1139)	0.0-0.3	＜0.001
2015	1.4 (25/1819)	0.9-2.0	0.1 (11157)	0.0-0.5	＜0.001
2016	1.8 (32/1771)	1.2-2.5	0.2 (3/1236)	0.0-0.7	＜0.001
2017	1.6 (28/1731)	1.1-2.3	0.2 (3/1239)	0.0-0.7	＜0.001
2018	1.4 (24/1753)	0.9-2.0	0.0 (0/1256)	0.0-0.3	＜0.001
2019	1.0 (17/1712)	0.6-1.8	0.0 (0/1243)	0.0-0.3	＜0.001
Total	1.1 (171/15742)	0.9-1.3	0.1 (7/10338)	0.0-0.1	＜0.001

Table S2. Overview of MCRPKP positive cases

No. of samples	Year	Gender	Age	Specimen	Plasmid
M050	2012	--	--	--	IncI2
M117	2015	Male	38	Drainage fluid	IncI2
M077	2016	Male	50	Blood	IncI2
M107	2016	Female	60	Bile	IncI2
M145	2017	Male	54	Sputum	IncF
M146	2017	Male	67	Sputum	--
M160	2017	Female	48	Drainage fluid	IncX4

--: information missing.

Table S3. Antimicrobial susceptibility profiles of 171 clinical *mcr-1*-positive *E. coli* strains

Drug Class	Antimicrobial agents	MIC_{50} μg/mL	MIC_{90} μg/mL	Range μg/mL	%R	%I	%S
Polypeptide	Colistin	4	8	0.25-16	87.7	0.0	12.3
Aminoglycoside	Amikacin	≤2	＞64	≤2-＞64	14.6	0.0	85.4
	Gentamicin	＞16	＞16	≤1-＞16	59.9	1.8	38.3
	Tobramycin	8	＞16	≤1-＞16	42.8	19.1	38.2
β-lactams	Ampicillin	＞32	＞32	≤2-＞32	95.9	0.6	3.5
	Cefazolin	＞64	＞64	≤4-＞64	87.1	0.0	12.9
	Cefepime	8	＞64	≤1-＞64	57.3	14.0	28.6
	Ceftazidime	16	＞64	≤1-＞64	52.0	3.5	44.4
	Cefotetan	≤4	≤4	≤4-＞64	3.8	0.0	96.2
	Ceftriaxone	＞64	＞64	≤1-＞64	78.3	5.6	16.1
	Ertapenem	≤0.5	≤0.5	≤0.5-＞8	2.3	1.8	95.9
	Imipenem	≤1	≤1	≤1-＞16	1.7	0.0	98.2
	Aztreonam	16	＞64	≤1-＞64	64.9	0.0	35.1
β-lactams/ Inhibitor	Ampicillin-sulbactam	＞32	＞32	≤2-＞32	77.2	15.6	7.1
	Piperacillin-tazobactam	≤4	8	≤4-＞128	2.8	6.2	91.0
Fluoroquinolone	Ciprofloxacin	＞4	＞4	≤0.25-＞4	81.9	2.3	15.8
	Levofloxacin	＞8	＞8	≤0.25-＞8	74.3	4.1	21.6
Other	Trimethoprim-sulfamethoxazole	＞256	＞256	≤32-＞256	71.2	0.0	28.8
	Nitrofurantoin	32	128	≤16-＞512	18.8	27.3	53.9

Table S4. Antimicrobial susceptibility profiles of 7 clinical *mcr-1*-positive *K. pneumoniae* strains.

No. of samples	Year	MIC (μg/mL)									
		**CL**	**AMK**	**GEN**	**TOB**	**AMP**	**CZO**	**FEP**	**CAZ**	**CTT**	**CRO**
M050	2012	6	≤2	>16	>16	>32	>64	>64	16	≤4	>64
M117	2015	12	≤2	>16	8	>32	>64	≤1	≤1	≤4	16
M077	2016	6	≤2	≤1	8	>32	≤4	≤1	≤1	≤4	≤1
M107	2016	12	≤2	≤1	>16	>32	>64	>64	>64	>64	>64
M145	2017	12	≤2	≤1	>16	>32	>64	>64	>64	>64	>64
M146	2017	12	≤2	≤1	≤1	>32	>64	>64	>64	>64	>64
M160	2017	12	≤2	>16	8	>32	>64	2	2	≤4	>64

No. of samples	Year	MIC (μg/mL)								
		**ETP**	**IPM**	**ATM**	**SAM**	**TZP**	**CIP**	**LEV**	**SXT**	**NIT**
M050	2012	≤0.5	≤1	16	>32	≤4	>4	>8	>256	256
M117	2015	≤0.5	≤1	≤1	>32	≤4	>4	>8	>256	128
M077	2016	≤0.5	≤1	≤1	>32	≤4	≤0.25	≤0.25	≤32	64
M107	2016	≤0.5	≤1	>64	>32	≤4	0.5	1	≤32	≤16
M145	2017	1	≤1	>64	8	≤4	≤0.25	≤0.25	≤32	32
M146	2017	>8	>16	>64	>32	>128	>4	>8	≤32	>512
M160	2017	≤0.5	≤1	16	>32	≤4	2	1	>256	≤16

*Antimicrobial drugs abbreviation: CL: colistin; AMK: amikacin; GEN: gentamicin; TOB: tobramycin; AMP: ampicillin; CZO: cefazolin; FEP: cefepime; CAZ: ceftazidime; CTT: cefotetan; CRO: ceftriaxone; ETP: ertapenem; IPM: imipenem; ATM: aztreonam; SAM: ampicillin-sulbactam; TZP: piperacillin-tazobactam; CIP: ciprofloxacin; LEV: levofloxacin; SXT: trimethoprim-sulfamethoxazole; NIT: nitrofurantoin.

Table S5. MIC values of *mcr-1*-positive *E. coli* from inpatients and outpatients.

Antimicrobial agents	Inpatient (n=124)			Outpatient (n=44)		
	Range	MIC50	MIC90	Range	MIC50	MIC90
Colistin	0.25-16	6	12	0.25-8	6	8
Amikacin	≤2->64	≤2	>64	≤2->64	≤2	4
Gentamicin	≤1->16	>16	>16	≤1->16	≤1	>16
Tobramycin	≤1->16	8	>16	≤1->16	4	>16
Ampicillin	≤2->32	>32	>32	≤2->32	>32	>32
Cefazolin	≤4->64	>64	>64	≤4->64	>64	>64
Cefepime	≤1->64	8	>64	≤1->64	8	>64
Ceftazidime	≤1->64	16	>64	≤1->64	16	>64
Cefotetan	≤4->64	≤4	≤4	≤4->64	≤4	8
Ceftriaxone	≤1->64	>64	>64	≤1->64	>64	>64
Ertapenem	≤0.5->8	≤0.5	≤0.5	≤0.5->2	≤0.5	≤0.5
Imipenem	≤1->16	≤1	≤1	≤1	≤1	≤1
Aztreonam	≤1->64	16	>64	≤1->64	16	>64
Ampicillin-sulbactam	≤2->32	>32	>32	≤2->32	>32	>32
Piperacillin-tazobactam	≤4->128	≤4	8	≤4->128	≤4	32
Ciprofloxacin	≤0.25->4	>4	>4	≤0.25->4	>4	>4
Levofloxacin	≤0.25->8	>8	>8	≤0.25->8	>8	>8
Trimethoprim-sulfamethoxazole	≤32->256	>256	>256	≤32->256	>256	>256
Nitrofurantoin	≤16->258	32	128	≤16->512	32	128

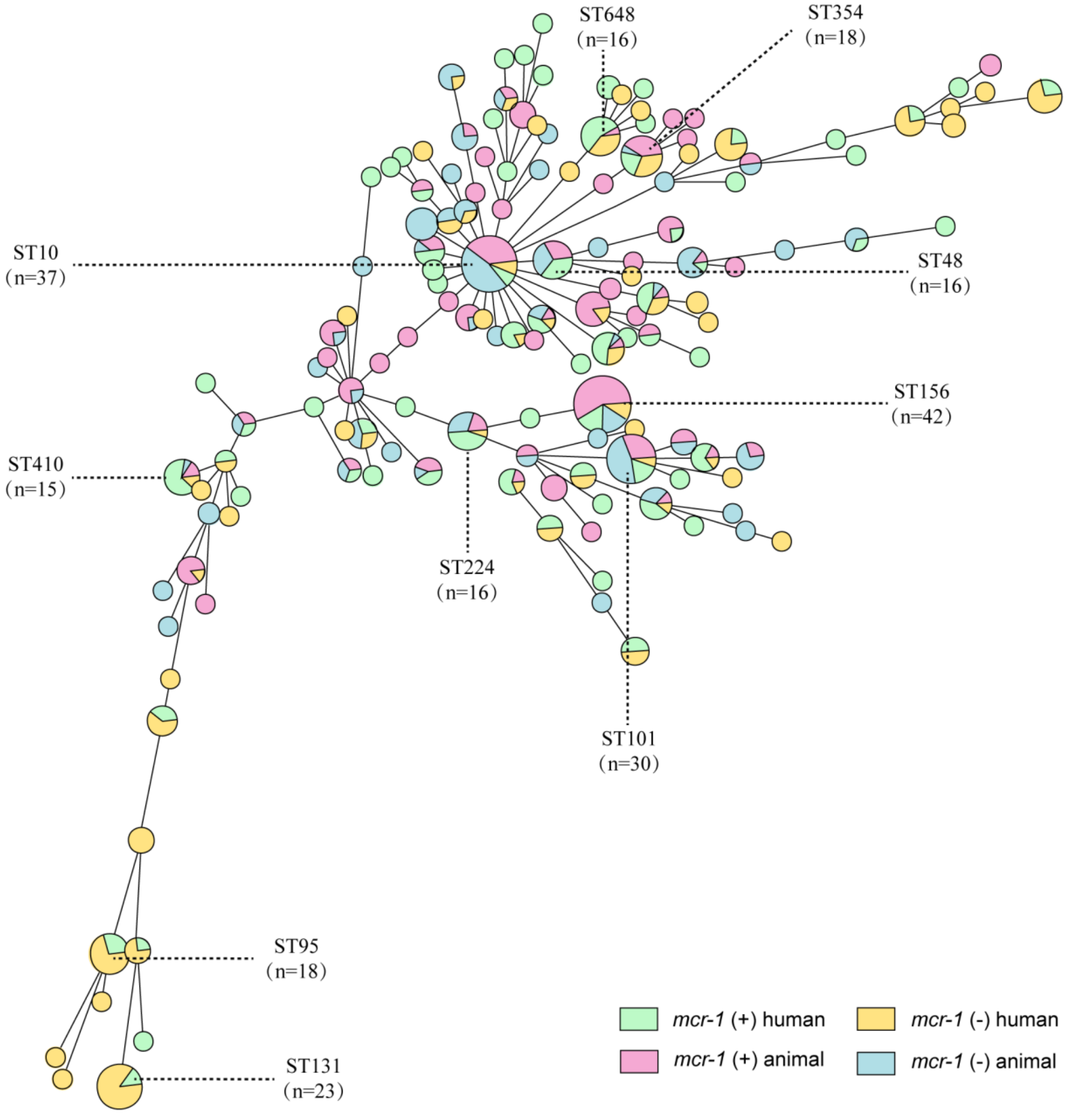

Fig. S1 Minimum spanning trees of mcr-1-positive *E. coli* by multilocus sequence type. Each node in the tree represents an ST, and the size of a node is proportional to the number of isolates it represents. The length of the branch is equal to the number of different alleles (calculated using seven MLST genes) between two linked nodes.

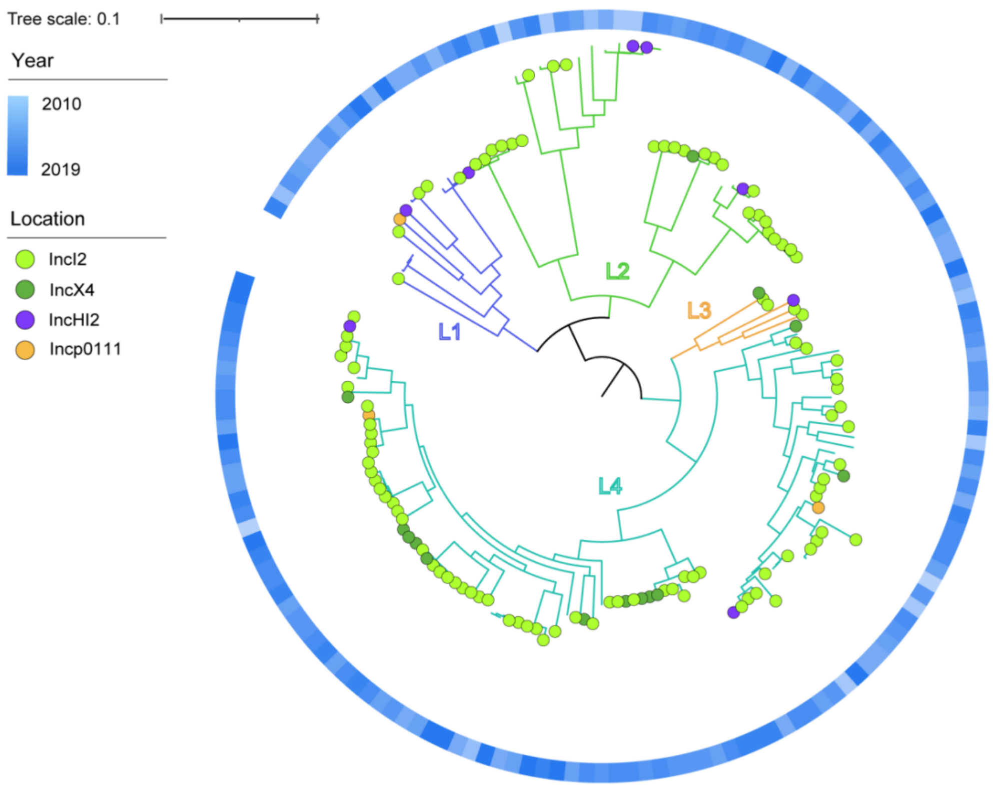

Fig. S2 Phylogenetic tree of all mcr-1-positive *E. coli* isolates from 2010 to 2019. The tree was conducted by core genome single nucletide polymorphism (SNP). Colored dots indicate the Inc type of the *mcr-1*-harboring plasmid (green, IncI2; dark green, IncX4; purple, IncHI2; orange, Incp0111).

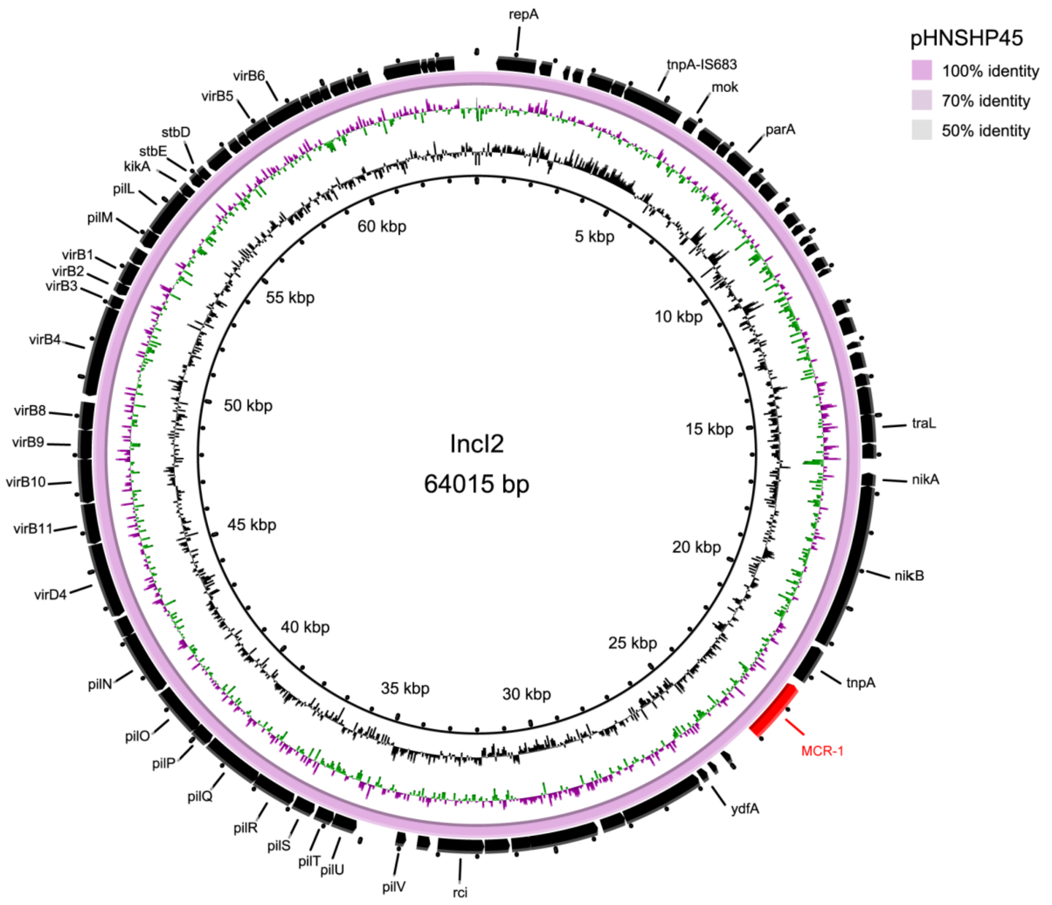

Fig. S3 Circular alignments of reference plasmid pHNSHP45 (accession number KP347127) sequences with IncI2 contigs from MCRPEC isolated in this study.

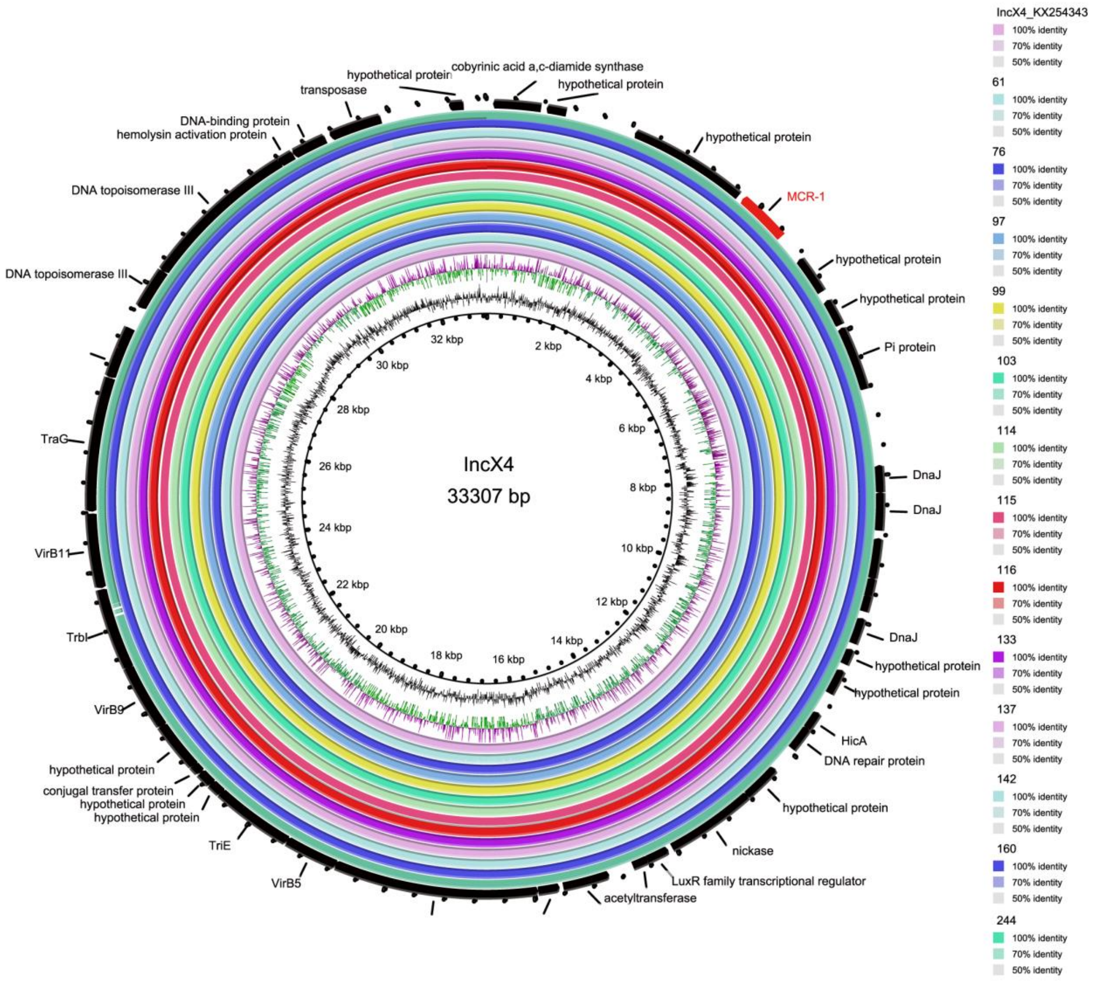

Fig. S4 Circular alignments of reference plasmid pECGD-8-33 (accession number KX254343) sequences with IncX4 contigs from MCRPEC isolated in this study.

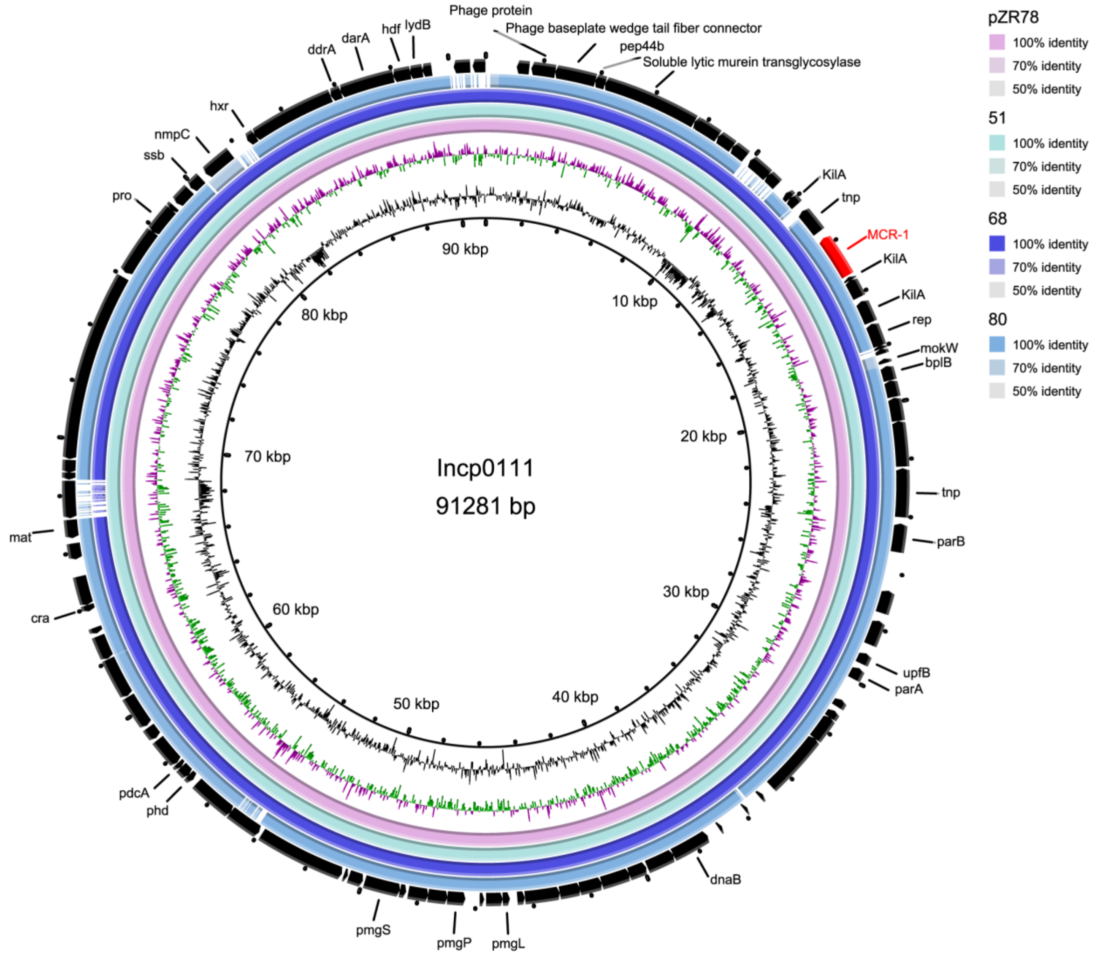

Fig. S5 Circular alignments of reference plasmid pZR78 (accession number MF455226) sequences with Incp0111 contigs from MCRPEC isolated in this study.

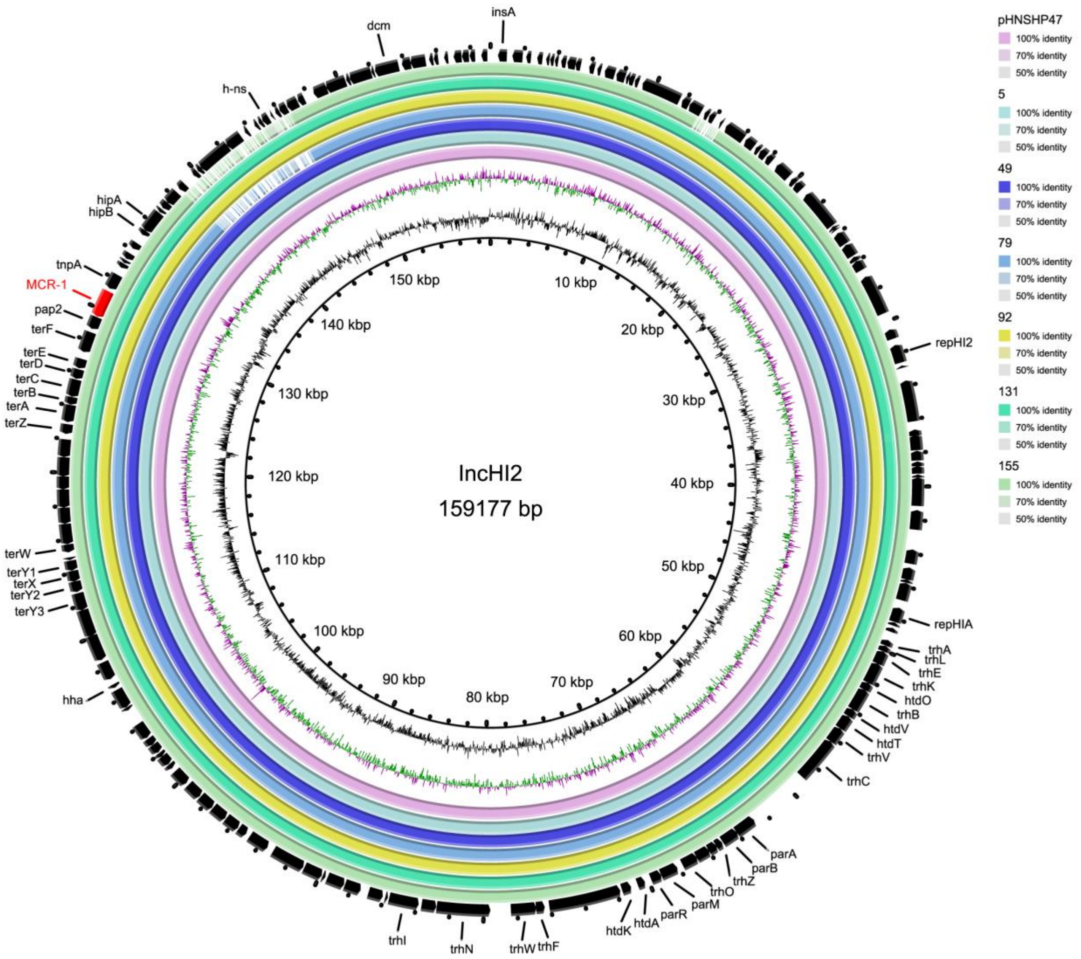

Fig. S6 Circular alignments of reference plasmid pHNSHP47 (accession number MF774186) sequences with IncHI2 contigs from MCRPEC isolated in this study.
